# A Case Report of Multiple Gastrointestinal Stromal Tumors: Imaging Findings, Surgical Approach, and Review of the Literature

**DOI:** 10.3389/fsurg.2022.886135

**Published:** 2022-05-20

**Authors:** Mariarita Tarallo, Cristina Carruezzo, Filippo Maria Dentice Di Accadia, Antonella Del Gaudio, Damiano Caruso, Michela Polici, Daniele Crocetti, Umberto Costi, Andrea Polistena, Francesco Panzuto, Andrea Laghi, Giuseppe Cavallaro, Enrico Fiori

**Affiliations:** ^1^ Department of Surgery Pietro Valdoni, University of Rome Sapienza, Rome, Italy; ^2^ Department of Medical Surgical Sciences and Translational Medicine, Radiology Unit, Sant’Andrea University Hospital, University of Rome Sapienza, Rome, Italy; ^3^ Department of Medical Surgical Sciences and Translational Medicine, Digestive Disease Unit, Sant'Andrea University Hospital, University of Rome Sapienza, Rome, Italy

**Keywords:** multiple gastrointestinal stromal tumors, gastrointestinal stromal tumor, computed tomography, surgical resection, minimally invasive surgery

## Abstract

**Introduction:**

Multiple gastrointestinal stromal tumors (GISTs) are rare tumors. Differential diagnosis between metastatic and multiple GISTs represents a challenge for a proper workup, prediction prognosis, and therapeutic strategy.

**Case presentation:**

We present the case of 67-year-old man with computed tomography (CT) evidence of multiple exophytic lesions in the abdomen, reaching diameters ranging from 1 to 9 cm, without any signs of organs infiltration, and resulting positive at 18F-FDG-PET/CT. Laparoscopic biopsy revealed multiple GISTs, and surgical resection by using an open approach was performed to achieve radicality. Moreover, an extensive review of the current literature was performed.

**Results:**

Small GISTs (<5 cm) can be treated by the laparoscopic approach, while in the case of large GISTs (>5 cm), tumor location and size should be taken into account to reach the stage of radical surgery avoiding tumor rupture. For metastatic GISTs, Imatinib represents the first choice of treatment, and surgery should be considered only in a few selected cases when all lesions are resectable.

**Conclusion:**

Sporadic multiple GISTs are a rare event, imaging findings are not specific for GISTs, and biopsy requires a secure diagnosis and proper management. In the case of large lesions, with a high risk of vessels injury, laparotomy excision should be considered to achieve radicality and to avoid tumor rupture.

## Introduction

Gastrointestinal stromal tumors (GISTs) are rare neoplasms (less than 1% of all gastrointestinal malignancies), representing the most common mesenchymal tumors of the gastrointestinal (GI) tract with an estimated annual incidence of 10–15 cases per million (1). GISTs usually appear as a single mass or, in rare occasions, as multiple lesions.

GISTs mostly occur in older individuals, having the median age of 55–65 years with a slightly male predominance (2). These tumors originate from the cells of Cajal, a site in muscularis propria and in the GI myenteric plexus, known as pacemaker cells of bowel peristalsis (3). The most common sites affected are the stomach (50%), small bowel (25%), colon-rectum (5%), and esophagus (<5%), and they may rarely occur in the omentum and mesentery (<5%), known as extra-GISTs (EGISTs) (4).

To date, initial diagnosis can be challenging because symptoms and signs are often mild and non-specific (e.g., nausea, vomiting, abdominal discomfort, and weight loss). However, in a few cases, GISTs can cause severe conditions such as bleeding, tumor rupture, dysphagia, and bowel or biliary obstruction (5). Severe symptoms are usually correlated to high-risk GIST according to Armed Forces Institute of Pathology risk classification, which is widely used to stratify patients (6, 7). According to the American Joint Committee of Cancer (AJCC) Staging Manual, 8th edition, tumor size, tumor location, and mitotic index are the key features correlated with patients’ prognosis (6–8).

Contrast enhanced computed tomography (CT) plays a primary role from diagnosis to follow-up; magnetic resonance imaging (MRI) represents a valid tool in selected cases (e.g., rectal GIST) (9). [18F]2-fluoro-2-deoxy-d-glucose (FDG) positron emission tomography (18F-FDG-PET/CT) represents a valid imaging modality to assess early response to therapy, restaging, and follow-up.

In order to select the best surgical approach, an accurate examination for multiple GISTs should consider the localization of the masses and the size of the greater lesion. The aim of our study is to present a case of multiple GISTs with a dedicated focus on imaging findings and surgical management.

## Case Presentation

We present a case of a 67-year-old man with a past medical history of hypertension, hyperuricemia, and left inguinal hernioplasty, who presented to the Emergency Department with a new onset of asthenia, abdominal pain, hypochromic stools, and hyperchromic urine.

His complete blood count was normal, except for elevated values of alanine aminotransferase (ALT), aspartate aminotransferase (AST), and gamma-glutamyl transferase (GGT).

### Imaging

**Figure 1:**
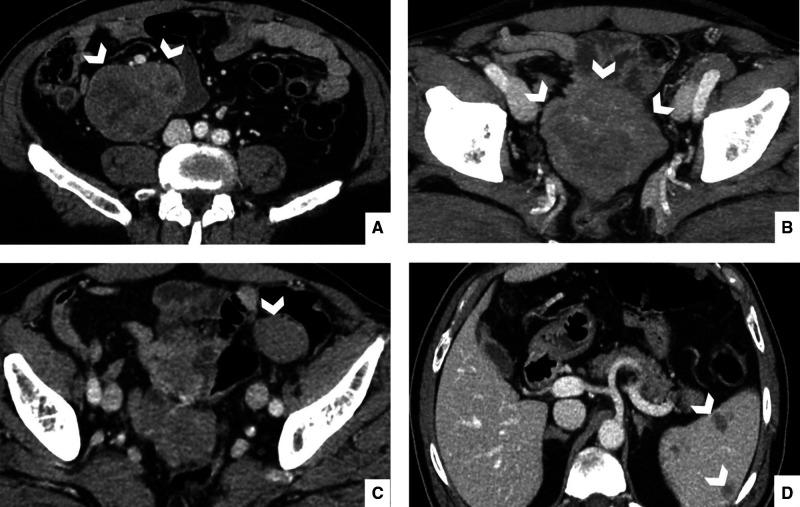
Portal phase, enhanced computed tomography of 67-year-old man, admitted to the Emergency Department with abdominal pain. Portal scans of the abdomen and pelvis shown in the right iliac fossa (**A**), a mass with moderate enhancement, measuring 7 cm, with hypodense areas. This mass had well-defined margins and appeared in contact with the ilium and vessels, but it did not infiltrate them. Another similar mass (**B**) appeared in the pelvic cavity in contact with the ileum; it measured a maximum diameter of 9 cm; this mass was also expansive but did not infiltrate adjacent structures. Other multiple smaller masses (**C**) were found on the right and left flank with maximum diameters of 3 cm. Multiple nodular pericentimetric formations (**D**) were present in the spleen, which were slightly hypodense.

The patient had already undergone contrast enhanced abdomen CT scan with a dedicated and optimized protocol (10), revealing two mass-like lesions, with diameters of 7 and 9 cm in the right iliac fossa and in the pelvis, respectively. These were characterized by moderate enhancement, rare hypodense areas due to the presence of necrotic- or cystic-changes, with focal spots of hemorrhages, and well-defined margins (Figure 1). The growth pattern was manly exophytic, without any signs of infiltration of organs and structures such as the ileum and vessels, which are closely adjacent. Other multiple similar smaller lesions were found on the left flank, with maximum diameters of 3 cm, and a 1-cm mass was found in the epigastric region. Furthermore, multiple pericentimetric solid nodules altered spleen parenchyma, with a high suspicion of metastatic nature.

**Figure 2:**
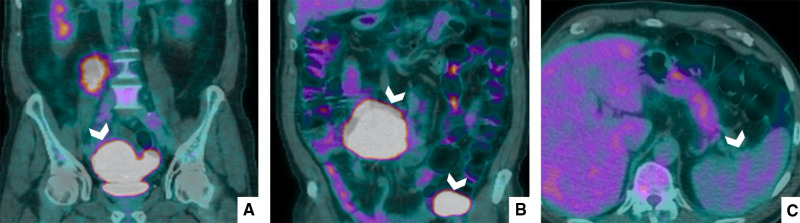
PET/CT with 18F-FDG. PET/CT shows that all lesions (**A**,**B**) had intense metabolic activity; however, the splenic nodular areas (**C**) did not pick up FDG.

The second step of diagnosis was 18F-FDG-PET/CT, in which almost all lesions showed a vivid uptake (SUV Max 7.3), reflecting an intense metabolic activity, with the exception of splenic lesions (Figure 2).

Then, for the final diagnosis, a biopsy of the pelvic mass was performed. Histological data demonstrated a neoplastic proliferation, with fibrous septa, showing a lobulated appearance, formed by cells with an elongated, hyperchromatic nucleus, with a proliferation index of more than 10%, without evidence of atypical mitosis; the cytoplasm was poorly definable and weakly eosinophilic. Neoplastic cells resulted positive for the reactions set up with anti-vimentin, anti-CD117, anti-CD99, anti-BCL2, and focally anti-EMA, while negative data emerged from the tests for antiprotein S100, antipancytokeratin, antismooth muscle actin, antidesmin, anti-CD34, anti-CD31, antipodoplanin, anti-HMB45, anticytokeratin 6/6, and anti-WT1.

### Surgical Technique

**Figure 3:**
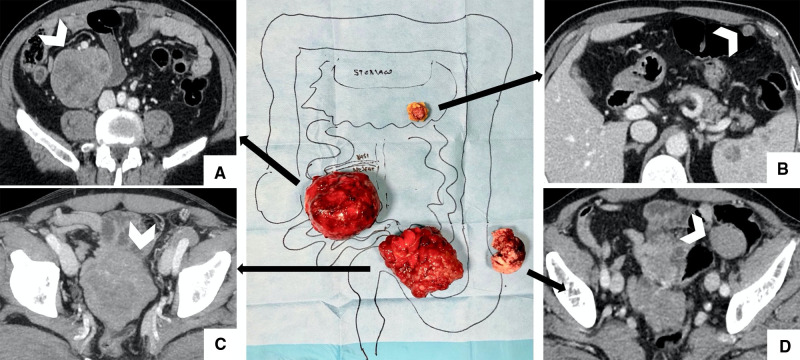
Surgical findings with imaging correlation. (**A**) Complete removal of the 7-cm mass is shown in the right iliac flank, close to the superior mesenteric vessels; (**B**) Centimetric omental mass was removed and analyzed by extemporaneous histological examination; (**C**) 9-cm mass (shown at CT in the pelvis) removed with segmental ileal loop resection to perform an en-bloc removal; (**D**) 4-cm mass in the left iliac region was completely removed.

At first, the patient underwent surgery with the laparoscopic technique. The exploration of abdominal cavity revealed a 7-cm mass in the right flank, close to the mesentery, a 4-cm mass in the left iliac fossa, and a smaller mass inside the omentum in the epigastric region. The omental mass was removed and analyzed by extemporaneous histological examination that reported a mesenchymal neoplasm. Consequently, complete surgical excision remained the main goal, and a midline laparotomy was carried out. During ileum mobilization, the 9-cm mass shown by CT was detected in the pelvis in tight contact with the ileum. The mass was mobilized with the intestinal loops and removed with segmental ileal loop resection. The 7-cm mass was shown close to the superior mesenteric vessels, so its complete removal required cautious handling. The mass on the left iliac region was also completely removed. All visible masses were completely removed, taking care to avoid tumor rupture (Figure 3). The definitive histological examination established that the diagnosis was multiple GISTs with spindle and epithelioid cells. Neoplastic cells were positive for DOG-1 and c-Kit. The marker of proliferation Ki-67 was 5%–10%.

### Follow-Up

The patient underwent adjuvant therapy with Imatinib. CT and PET/CT scan after 6 months showed a full resolution of the clinical picture, with no uptake of 18-FDG upon PET/CT scan. The patient is still receiving adjuvant therapy, and a CT scan is scheduled at 12 months’ time (6 months from the last one).

## Review of the Literature and Discussion

We described the case of a patient affected by multiple GISTs, with intense abdominal pain and hypochromic stools and hyperchromic urine, who underwent surgical procedure upon admission in the Emergency Department. Our intent was to achieve the radical resection of all lesions without considering the neoadjuvant therapeutic option due to the emergency and symptomatic presentation of the patient. Postoperative histological examination and mutational panel analysis revealed multiple GISTs.

GISTs are usually solitary lesions, but in extremely rare cases, multiple GISTs may be detected in one or more organs. Single GISTs are often characterized by good prognosis in comparison with multiple GISTs, according to recent evidence (11).

With regard to radiologic findings, GISTs are usually represented as heterogeneous-enhancing mesenteric lesions with hemorrhagic or necrosis areas or cystic components. Many lesions present as isolated masses in the mesentery entering the differential diagnosis of GISTs, in particular: desmoid fibromatosis (DF), sclerosis mesenteritis (SM), lymphoma, neuroendocrine tumor (NET), and liposarcoma. DF is a soft tissue mass mainly composed of mesenchymal tissue. Upon unenhanced CT, it has a homogenous density and enhances slightly heterogeneously with intravenous contrast. SM presents with the characteristic “Halo sign”, a hyperdense mass with surrounding mesenteric vessels and soft tissue nodules, and frequently contains calcifications. A pseudocapsule can be present, with a dense band with normal fat surrounding the inflamed lesion. The classical CT appearance of mesenteric lymphoma is a “sandwich” appearance: an extensive homogeneously enhancing lymphadenopathy and occasional central necrosis enveloping the mesenteric vessels with preserved perivascular fat borders. Usually, NETs are hypervascular lesions with fibrous adhesive bands with outward radiating peritumoral vessels and contain coarse calcifications. Liposarcoma generally shows a non-homogeneous lesion with fat density intermixed with zones of denser tissue; the borders are poorly defined and often infiltrating. Contrast uptake is heterogeneous and progressive (12, 13).

One of the main challenges is the differential diagnosis of multiple sporadic GISTs when non-syndromic, which are often misdiagnosed as metastatic GISTs (14). In fact, multiple GISTs could often occur in the case of Carney’s triad, Carney–Stratakis syndrome, and neurofibromatosis type I (15, 16); nevertheless, multicentricity alone is not an evidence of a syndromic or hereditary disease (17). In multiple GISTs, each lesion is characterized by different mutational panels, while metastatic GISTs show the same genetic pattern in all lesions. In fact, some authors state that a genetic analysis of KIT/PDGFRA could be helpful for differential diagnosis (17, 18).

To date, some authors have published their personal experiences in the form of case reports or small case series of multiple GISTs. Shen et al. describe how they classified patients based on genetic analysis of the specimen, starting from 44 initial diagnosis of multiple GISTs to 27 truly multiple sporadic GISTs. They performed mutation analysis with genomic DNA isolated from formalin-fixed paraffin-embedded tissues; all patients underwent surgery with the removal of all lesions (19). Li et al. state that radical surgery is the primary treatment for multiple GISTs, and that it is important to evaluate the number and sites of tumors for the removal of all lesions. They reported a personal record of 20 patients affected by multiple GISTs (17).

The current guidelines do not define a structured workflow for these patients (9) due the limited scientific evidence about multiple sporadic GISTs (19). However, a surgical procedure with a radical approach may be considered as a first treatment option for multiple non-metastatic GISTs. However, Imatinib, a tyrosine kinase inhibitor of KIT/PDGFRA, is considered the first option for metastatic and/or unresectable GISTs (20).

According to the size and the number of lesions, GISTs are categorized into three groups: small GISTs, large GISTs, and metastatic GISTs (21). In general, small GISTs (<5 cm) are resected with R0 pathology results, without the need for lymphadenectomy, also in consideration of expansive growth and the extremely rare occurrence of nodal metastasis. Today, the laparoscopic approach can be considered the standard procedure, because it has proved to be less invasive and is characterized by shorter postoperative time of recovery in comparison with conventional laparotomy (21). Recently, Nishida et al. reviewed the literature with the aim of summing up the main guidelines about the treatment of small GISTs, and they observed that there is no global consensus (21). In fact, Japanese and Asian GIST guidelines recommend surgical resection for gastric GISTs <2 cm (22, 23), while the National Comprehensive Cancer Network (NCCN) guidelines recommend surgical resection only for small gastric GISTs with high-risk features (24). However, all the main guidelines, including the NCCN and ESMO guidelines (1, 24), suggest the surgical approach for treating small rectal GISTs due to the different clinical behaviors and prognostic outcomes.

Large GISTs are considered tumors bigger than 5 cm, and complete surgical excision should be considered (9, 24). The main goal of surgery is to achieve radicality without the risk of tumor rupture, representing an adverse prognostic factor (25). In such a scenario, laparoscopy can also be performed for large GISTs, but it can be performed with accurate surgical planning by considering tumor location and size, with a specific focus on tumors greater than 8 cm, for which the chance to use the laparoscopic approach seems to be limited (21). In case of large GISTs with an infiltrating growth pattern, surgical resection is recommended with the object to preserve as much as possible the functionality of the organs involved (26).

When GISTs are not eligible for radical surgery, neoadjuvant target therapy, Imatinib, could be considered as a chance to reduce the size of tumors (27). The group of Şentürk demonstrated in 151 GIST patients that radical resection of the tumor is the ideal treatment, while Imatinib therapy should be administered in large GISTs considered unresectable (28). With regard to metastatic GISTs, Imatinib represents the first option, while surgery should be considered only in a few selected cases when all lesions are resectable (21, 27, 29). The introduction of the tyrosine kinase inhibitor in the management of GISTs has radically improved the outcome of patients with high disease burden (30). In case of unresectable metastatic disease, target therapy should be continued indefinitely (9).

With regard to follow-up, there is no consensus on the optimal routine for patients affected by GISTs. Programs differ across institutions. The last updated guidelines reported that high-risk patients should undergo abdominal CT scan or MRI every 3–6 months for 3 years during adjuvant therapy, then every 3 months for 2 years (on cessation of adjuvant therapy), then every 6 months until 5 years, and annually for an additional 5 years; low-risk patients should undergo abdominal CT scan or MRI every 6–12 months for 5 years. (9).

## Conclusion

Multiple GISTs need to be differentiated from metastatic GISTs for ensuring the appropriate therapeutic management and outcome prediction. The surgical approach may be considered as the first treatment option for multiple GISTs, whereas Imatinib is mandatory for metastatic GISTs. According to the guidelines, small GISTs (<5 cm) can be treated by laparoscopy, while for large GISTs, the surgical approach should consider tumor location and size. For multiple GISTs, the dimensions of the lesions should be evaluated; we suggest surgical planning with a specific focus on the biggest lesion. In our case, after a first consideration of the laparoscopic approach, a conversion to laparotomy was chosen due to lesion dimensions and the close relation of the lesions with the vessels, to avoid tumor rupture.

## Data Availability

The raw data supporting the conclusions of this article will be made available by the authors, without undue reservation.
